# Understanding trait impressions from faces

**DOI:** 10.1111/bjop.12583

**Published:** 2022-07-26

**Authors:** Clare A. M. Sutherland, Andrew W. Young

**Affiliations:** ^1^ School of Psychology, King's College University of Aberdeen Aberdeen UK; ^2^ School of Psychological Science University of Western Australia Crawley Western Australia Australia; ^3^ Department of Psychology University of York York UK

**Keywords:** data‐driven approaches, face perception, first impressions, social cognition models, trait attributions

## Abstract

Impressions from faces are made remarkably quickly and they can underpin behaviour in a wide variety of social contexts. Over the last decade many studies have sought to trace the links between facial cues and social perception and behaviour. One such body of work has shown clear overlap between the fields of face perception and social stereotyping by demonstrating a role for conceptual stereotypes in impression formation from faces. We integrate these results involving conceptual influences on impressions with another substantial body of research in visual cognition which demonstrates that much of the variance in impressions can be predicted from perceptual, data‐driven models using physical cues in face images. We relate this discussion to the phylogenetic, cultural, individual and developmental origins of facial impressions and define priority research questions for the field including investigating non‐WEIRD cultures, tracking the developmental trajectory of impressions and determining the malleability of impression formation.

## INTRODUCTION

**FIGURE 1:**
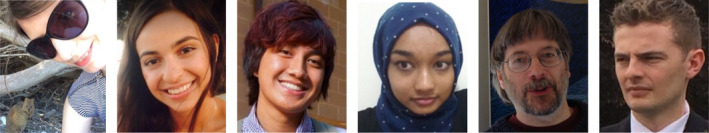
Whether walking down the street or browsing online, our visual systems are exposed to a vast range of facial cues, as illustrated by these example photographs. First impressions can be made easily and intuitively from naturalistic images such as these

People form impressions of a stranger's character in a fraction of a second (Henss, 1991; Willis & Todorov, 2006), in spite of the common advice ‘not to judge a book by its cover’ (Figure 1). Many studies have shown that people tend to agree on their impressions and that impressions from faces are formed readily (Klapper et al., 2016; Oosterhof & Todorov, 2008), implicitly (Swe et al., 2020) and with minimal information; even a single glimpse of a photograph can suffice (Willis & Todorov, 2006). Impressions of traits from faces have at best only limited accuracy, meaning that their everyday use is problematic (Foo et al., 2021). Yet, despite this limited accuracy, facial impressions often have critical social consequences. For example, perceived facial trustworthiness can predict consumer choices (Ert et al., 2016), online financial lending (Duarte et al., 2012) and even death penalty decisions (Wilson & Rule, 2015). Moreover, these impressions are often hard to shift (Chang et al., 2010) and can affect social behaviour even in the presence of more valid cues (Ert et al., 2016). Unfortunately, with the rise of social media and online peer‐to‐peer marketplaces, the pervasive influence of impressions from face images is likely increasing (Sutherland, Burton, et al., 2020). Given the importance of facial impressions to everyday decisions, it is crucial that we understand how and why they are formed.

Here, we aim to further this understanding by providing an overarching perspective on key questions that have been addressed by the field, as well as outlining future research priorities. We discuss how visual cues, social stereotypes and cultural and individual differences have all been shown to contribute to impressions from faces. We also discuss the insights given by social learning and evolutionary accounts of impression formation. In doing so, we show how impressions from faces relate to wider theoretical concepts in face and person perception.

### How are facial impressions formed?

Impressions are famously rich: for example, nearly 18,000 words in English refer to descriptions of others' personality and behaviour (Allport & Odbert, 1936), while faces contain a wealth of visual and social cues to impressions (Bruce & Young, 2012; Calder & Rhodes, 2011). Moreover, impressions themselves are often intercorrelated, rendering it difficult to disentangle which impressions are driving any real‐world consequences or to build a complete theory of facial impression formation (Secord, 1958; Todorov et al., 2015). For example, impressions of trustworthiness are highly correlated with impressions of niceness and warmth (Collova et al., 2019).

Recently developed data‐driven approaches to facial impressions (Box 1) have proved to be fruitful, because they take advantage of this multicollinearity to model key dimensions underlying a range of impressions from face photographs. In this approach, principal components analysis or factor analysis is used to reduce a wide variety of impressions into a smaller set of dimensions which capture underlying patterns of covariation among those impressions. By creating a model based on dimensions rather than specific traits, the approach can elegantly capture key sources of variation across many different trait impressions; the resulting dimensional structure can also give insights into how such a wide range of impressions can be formed from photographs of faces. It is important to be clear that these dimensional trait models are created only from social impression judgements; although they may be influenced by physical cues in faces (section ‘Visual cues to face impressions’) they are conceptually and empirically distinct from the faces themselves (Todorov & Oh, 2021).

BOX 1 **Data‐driven methods in vision science**

Data‐driven methods have increasingly gained in importance in vision research (Adolphs et al., 2016) because they possess the considerable advantage that they minimize the extent to which an investigation becomes overly constrained by any one *a priori* hypothesis. Specific data‐driven methods vary, but all share the core feature that, rather than testing one cue at a time, a wide variety of potential visual cues are sampled at once, either by randomly generating faces using computer‐generated methods (Abir et al., 2017; Jack & Schyns, 2017; Oosterhof & Todorov, 2008), by introducing random noise patterns superimposed on faces (Dotsch et al., 2008) or by sampling real‐world, natural variation with naturalistic photographs of faces (Sutherland et al., 2013).

Data‐driven methods are especially helpful when investigating complex, multifaceted stimuli and concepts like face images and/or trait representations. In the case of face perception, this advantage arises because data‐driven methods can sample many facial cues simultaneously (Todorov, 2017; Vernon et al., 2014). In contrast, 1024 conditions would be needed to independently manipulate just 10 (binary) facial features. With more realistic manipulations, the number of conditions needed can easily reach the high thousands, an approach that is clearly unwieldy (Todorov, 2017). Moreover, data‐driven methods can give unique insights into which visual cues are especially important relative to others, or important in combination (Sutherland et al., 2013) and are especially useful in understanding cultural variation while minimizing top‐down biases (Jack et al., 2018; Sutherland et al., 2018). Finally, data‐driven methods may be particularly appropriate to investigate impressions from faces precisely because these are driven by a holistic combination of multiple cues (Vernon et al., 2014). For example, smiling may look confident or appeasing depending on head tilt and gender (Vernon et al., 2014).

Although they offer substantial benefits, data‐driven approaches do have limitations. Although typically very rich, the findings are not constrained by specific hypotheses; it can therefore be helpful to run complementary studies using more conventional methods to support or refute hypotheses derived from the data‐driven patterns. Moreover, the conclusions drawn are highly sensitive to sampled variation (e.g. strong conclusions about how people form impressions from Asian faces cannot be drawn from models which do not sample Asian faces: Sutherland et al. 2018), although generalizability is clearly also important for hypothesis‐driven research. Thus, it is highly important to seek to minimize biases in image selection in data‐driven studies: for example, it is important to check that datasets are representative of the population they are drawn from, ideally by asking participants or researchers from that region or social group to verify the generalizability of images or meaning of verbal or other qualitative descriptions, and by taking images from representative sources (e.g. from Chinese as well as UK search engines for images retrieved online; Sutherland et al. 2018). Finally, these methods are limited in their ability to isolate the effects of specific cues. Of course, it is possible that the meaning of each cue can change in the context of other cues, making it impossible to find a template that captures all meaningful variation (see Sutherland et al., 2014). Ultimately, however, whatever cues are revealed in a data‐driven approach is necessarily a function of the face stimuli and the raters; capturing the multiplicative nature of cues is a challenge that might be resolved only with respect to a specific set of facial stimuli. Overall, data‐driven models appear to work best at the theory‐generation side of science and to lead to new hypotheses to test (e.g. about which specific facial cues are important in forming impressions).

Although data‐driven methods have mainly been used in visual perception in the last decade (Adolphs et al., 2016), it is important to recognize that these methods have long been used to answer fundamental questions in social cognition and personality research (Fiske & Macrae, 2012). In particular, data‐driven methods lie behind the development of models of stereotypes (Fiske et al., 2007), attitudes (Osgood, 1969) and personality (Rosenberg et al., 1968), including the Big Five (Goldberg, 1990). Across fields, data‐driven approaches share a focus on *modelling* rather than *reducing* variation, and an interest in understanding broad underlying patterns.

Data‐driven dimensional approaches have been very influential over the last decade, inspired by Todorov's original valence and dominance model of impressions (Oosterhof & Todorov, 2008). These approaches have revealed that a small number of dimensions (two to four) can account for most of the variance in facial impressions (Lin et al., 2021; Oosterhof & Todorov, 2008; Sutherland et al., 2013; Walker & Vetter, 2016; Wolffhechel et al., 2014). Across studies, the first dimension, which explains most variance (around 30%–60%), consistently seems to involve traits related to judging someone's good or bad intentions (hence Oosterhof & Todorov, 2008’s original ‘valence’ label). Traits which contribute to this dimension include trustworthiness (Jones et al., 2021; Oh et al., 2020; Oosterhof & Todorov, 2008), interpersonal warmth (Lin et al., 2021; Walker & Vetter, 2016), niceness (Collova et al., 2019) and approachability (Sutherland et al., 2013; Wang et al., 2019). Facial cues which lead to impressions of good intentionality include smiling, older age, head tilt and femininity along with female gender (Oosterhof & Todorov, 2008; Vernon et al., 2014).

An open question regards the extent to which different facets of good/bad intentionality are related. One study found that morality‐related impressions from faces (e.g. trustworthiness) can be dissociated from sociability‐related impressions (e.g. warmth: Oliveira et al., 2020; see also Lin et al., 2021), capturing the intuition that ‘one may smile and smile and yet be a villain’ (Hamlet, 105–109). However, other studies find a close relationship between these aspects of intentionality as judged from faces (Sutherland, Oldmeadow, et al., 2016; Walker & Vetter, 2016). For example, using a reverse correlation approach, Oliveira and colleagues showed high overlap between warmth and trustworthiness in both facial features and in their relationship with valence (Oliveira et al., 2019). This question of whether morality and sociability can dissociate parallels a longstanding debate in social psychology from studies where impressions are made from vignettes, real‐life text (e.g. obituaries) or personal recollections (Brambilla et al., 2011; Fiske et al., 2007; Goodwin et al., 2014). We suggest that the extent to which different facets of intentionality emerge may depend on the cues available to the perceiver: face‐based impressions (especially from single photographs with strong emotional or other naturalistic cues) are likely more superficial and thus less differentiated than those based on behaviour, familiarity with a person, or where stimuli are more tightly controlled (Sutherland, Rowley et al., 2015). Definitions may also be important; for example, Lin et al. (2021) did not find that trustworthiness related strongly to their first, warmth dimension, when trustworthiness was defined for participants as ‘A person who can be relied on as honest and truthful’. In other studies, however, participants are often left to determine their own interpretation of trait labels and may construe trustworthiness more broadly as friendliness or approachability; individual differences in trait construal are also likely to be important (see section ‘Origins in individual learning’). Words used to describe trait concepts could also differ in meaning depending on the context in which they are used: when applied specifically to judgements of people from faces, these words need not mean the same thing as when referring to personality or behaviour in the abstract (although they can correspond; see section ‘Social stereotype cues’).

Most studies also find a separate face‐based dimension corresponding to traits related to capability in carrying out one's intentions, including dominance (Jones et al., 2021; Oh et al., 2020; Oosterhof & Todorov, 2008; Sutherland et al., 2013), competence (Lin et al., 2021; Sutherland et al., 2018; Wang et al., 2019) agency (Walker & Vetter, 2016) or status (South Palomares et al., 2018). Facial cues to capability typically include age, masculinity, attractiveness and confident expression (Oh et al., 2019; Vernon et al., 2014). In general, dimensions related to capability explain less variation in impression formation (around 10%–30%) than those primarily related to judging intentions. Moreover, traits on this dimension are more variable than on the intentionality dimension, as physical‐based capability (dominance) has been found to diverge from social‐based capability (competence: Lin et al., 2021; Oliveira et al., 2019, 2020; Sutherland, Oldmeadow, et al., 2016). In part this divergence might be because dominance can be perceived negatively, especially for female faces, while competence is perceived positively (Oliveira et al., 2019, 2020; Sutherland, Young, et al., 2015). We have suggested that traits along this capability dimension may also be inherently contextual, given that capability is specific to the task or domain at hand (Sutherland, Oldmeadow, et al., 2016).

Several studies also find a dimension corresponding to age, along with associated stereotypes including (decreased) attractiveness and increased babyfacedness. Perhaps unsurprisingly, an age‐related dimension appears to be more likely to be found where there is variation in the age of the stimulus faces (Lin et al., 2021; South Palomares et al., 2018; Sutherland et al., 2013; although sometimes it appears with younger faces too; Wolffhechel et al., 2014), again highlighting the importance of stimulus image variability to the data‐driven approach. This dimension appears to explain around 10%–20% of the variation in impressions. Traits along this youthful‐attractiveness dimension may be related to romantic partner preferences, age stereotypes or simply a general judgement of health. Indeed, romantic partner preferences are most strongly predicted by traits along this factor, at least for heterosexual, young adult student perceivers (South Palomares & Young, 2019).

Finally, one recent study found that impressions related to gender (masculinity versus femininity) can form a fourth dimension when more traits are sampled (Lin et al., 2021). A similar dimension also appears sometimes at the individual participant level (Sutherland, Rhodes, et al., 2020), and may be linked to the finding that physical capability or dominance, which is closely linked to masculinity, can diverge from social capability, as discussed before.

Although these impressions will be to some degree dependent on the faces, words/labels and experimental paradigms used, and there are key differences across studies, likely due to important face and trait input variation (see Box 1 and sections ‘Origins in individual learning’ and ‘Evolutionary origins’), it seems to us rather remarkable that substantial similarities appear across studies under these circumstances. Indeed, the underlying function of impression dimensions is surprisingly similar between studies using different faces (e.g. Oosterhof & Todorov, 2008; Walker & Vetter, 2016), labels (e.g. Sutherland et al., 2013; Wolffhechel et al., 2014), and, as we shall see, also across different types of stimuli (e.g. McAleer et al. 2014, Hu et al. 2018 Osgood, 1969), and approaches (e.g. dimensionality reduction versus cluster analysis or multidimensional scaling: Rosenberg et al. 1968). Most interestingly, across all studies, just 2–4 trait dimensions appear to account for around 70%–80% of the variance in impressions of face images studied so far (Oosterhof & Todorov, 2008; South Palomares et al., 2018; Sutherland et al., 2013; Walker & Vetter, 2016).

#### Visual cues to face impressions

A key insight has been the finding that it is possible to predict complex first impressions simply by measuring visual properties of photographic images. For example, Vernon and colleagues found that neural network models trained purely on 65 physical face features from everyday face photographs were able to predict more than half the variance in social impressions from new (untrained) face images (Vernon et al., 2014). In Vernon et al.’s study these visual attributes were marked up by hand, but more recent work has shown that visual image statistics based only on low‐level shape and texture properties can predict observers' consensual impressions of trustworthiness, attractiveness and dominance to a comparable extent (Mileva et al., 2019). Data‐driven models have also been used to compare the contributions of different visual cues to attractiveness, an approach which can be extended to other traits (Holzleitner et al., 2019); and Oosterhof and Todorov (2008) seminal work also included a computational model of the physical facial features which lead to impressions. Finally, deep neural networks created to mimic some aspects of primate visual cortex, and trained to recognize face identity, can also predict social trait judgements from face images (Parde et al., 2019), again suggesting a visual underpinning to impression judgements. Note that these visual models are conceptually and empirically distinct from social impression models, being based on completely different data (i.e. physical cues measured in images themselves, instead of trait judgements). Thus, any correspondence between social and visual models is highly interesting (Vernon et al. 2014).

It was an important step to be able to explain such a substantial proportion of the variance in impressions from everyday images, but can we say more about which visual attributes actually support these impressions? A useful way to think about these visual impression cues is that they fall under three general categories: invariant, changeable and environmental (Sutherland, Young, et al., 2016).

Relatively invariant visual cues within the face include the person's unique facial identity, as well as social category information such as ethnicity, gender and age, as signalled through a combination of physiognomy and skin tone (e.g. Mileva et al., 2019; Oosterhof & Todorov, 2008; Zebrowitz & Montepare, 1992). Changeable cues within the face include momentary characteristics such as emotional expression, head tilt, head orientation and gaze direction (e.g. Hehman et al., 2013; Sutherland, Young, et al., 2016; Torrance et al., 2020). Finally, impressions may be affected by changeable environmental cues outwith the face itself, including physical location, lighting, camera and photographic angle. Accessories such as hairstyle, facial hair, visible clothing, glasses or jewellery can also influence trait impressions (e.g. Oh et al., 2020; Pellegrini, 1973; Sutherland et al., 2013; Vernon et al., 2014).

These categories overlap and interact; for example, lightness and hue in skin tone varies by one's (relatively invariant) social group, across changing seasons and health, and, in a photograph, due to environmental changes in lighting, temperature, blushing, pose and make‐up. As another key example, changeable expression cues can signal identity, while structural facial cues in an expressionless face can be misattributed as emotional expression (Secord, 1958; Zebrowitz et al., 2010). Importantly, the overall impression of a face image is generated through a holistic combination of multiple cues (Santos & Young, 2011; Todorov et al., 2010). For example, trustworthiness is often associated with a combination of open smiling, femininity and healthy skin tone, whereas increased attractiveness involves a combination of decreased age, wide eyes, femininity and healthy skin tone (Vernon et al., 2014). These interactions mean that any search for the single ‘diagnostic’ cue that defines a particular type of impression will be of limited success. Indeed, the importance of cue combinations has long been recognized in social psychological studies on impressions (Secord, 1958).

The strong dependence of facial impressions on highly changeable (including environmental) cues alongside relatively invariant cues may help explain the initially surprising finding that impressions can differ as much based on the different photographs of the same people, as by different target people depicted (Collova et al., 2020, 2021; Jenkins et al., 2011; Sutherland, Young, et al., 2016; Todorov & Porter, 2014). It is a common misinterpretation to think that an impression based on a face image is the same thing as an impression of the face itself (a classic perceptual conundrum: ‘*ceci n'est pas une pipe’*). Understanding how facial and extra‐facial cues combine to create impressions is a priority for the field.

Despite the multitude of the cues involved, impressions from face images are made remarkably quickly, needing only a split‐second view (Willis & Todorov, 2006). Face‐based impressions have been claimed to be an automatic process and indeed do fulfil some criteria for automaticity (Willis & Todorov, 2006). Specifically, trait information can be extracted from faces quickly (Willis & Todorov, 2006), spontaneously (Klapper et al., 2016), unconsciously (Freeman et al., 2014; Getov et al., 2015) and attractiveness impressions, at least, may be mandatory (Ritchie et al., 2017). Recent electrophysiological studies using the fast periodic visual stimulation technique have also established that a neural signal can be found in response to changes in trustworthiness in rapidly presented face images, without requiring instructions to judge trustworthiness (Swe et al., 2020; Verosky et al., 2020). It has yet to be established whether face‐based impressions are automatic in the sense of being capacity‐free. Overall, though, impressions can be made remarkably easily from visual input, without apparently needing much cognitive elaboration.

#### Social stereotype cues

Although much of the variability in trait impressions can be explained from purely visual properties, theorists have also been interested in the extent to which conceptual stereotypes relate to these impressions. In the mid‐20th century, Secord first suggested that impressions can also be formed from contextual social inferences; for example, the inference that people who wear glasses are often clever (Secord, 1958). Indeed, people wearing glasses do look more intelligent (Leder et al., 2011; Sutherland et al., 2013). Stereotypes of social groups (for example, that nurses are generally healthy and nice) can also be found in impressions of faces (for example, faces that look healthier and nicer are thought to look more like nurses) (Oldmeadow et al., 2013).

**FIGURE 2:**
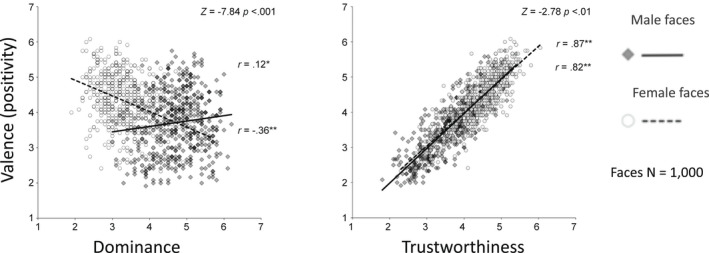
Different evaluative consequences of trait dominance impressions for male and female faces. Dominance in appearance is seen as having a slightly positive valence for men, but a negative valence for women. Trustworthiness, in contrast, is highly positively valenced for both genders. Data from Sutherland et al., 2015, *British J. of Psychology*

Moreover, while the same overall dimensions can account for impressions of both male and female faces (Oh et al., 2020; South Palomares et al., 2018), there are striking links between these facial impressions and face gender, which parallel conceptual gender stereotypes. For example, female faces are on average judged as more trustworthy than male faces, and male faces are judged as more dominant than female faces (Sutherland, Young, et al., 2015). Dominance itself is conveyed by stereotypically masculine cues, like a broad jaw (Oosterhof & Todorov, 2008; Sutherland et al., 2013). Moreover, the dominance dimension has different connotations across face gender: female facial dominance is viewed negatively, but male facial dominance is viewed as neutral, ambiguous or even as slightly positive (Figure 2: Oh et al., 2020; Oliveira et al., 2020; Sutherland, Young, et al., 2016). Importantly, differences in the connotations of traits are found across gender even when the faces are controlled for how much of that trait they express (e.g. for levels of rated trustworthiness across male and female faces), suggesting a contribution from evaluative stereotypes over and above physical sex differences per se. Although it remains possible that bottom‐up interactions between different trait and gender cues in faces may make an additional contribution, this pattern of ‘top down’ influences fits well with prevailing gender stereotypes, echoing wider prejudice seen against women who behave dominantly (Rudman et al., 2012).

The relative importance of the dimensions of face impressions also differs by face gender, so that youthful‐attractiveness explains more variation for female faces and capability (status) explains more variation for male faces (South Palomares et al., 2018). This pattern mirrors models of (heterosexual) conceptual partner preferences, in which men are found to prioritize attractiveness and women status, when thinking about ideal romantic partners (Fletcher et al., 2004). Finally, cues to competence also differ across women and men (Oh et al., 2019). Again, these differences echo prevailing *conceptual* stereotypes of men and women (Fiske, 2012; Stolier et al., 2017).

More generally, a striking overlap exists between models of facial impressions and models of stereotypes developed from purely verbal concepts. For example, the three main dimensions of facial impressions found by Sutherland et al. (2013) show strong parallels with tripartite models of verbally expressed romantic partner preferences (South Palomares et al., 2018). Moreover, the valence (trustworthiness) and dominance dimensions also seem to parallel warmth and competence in the Stereotype Content Model, often labelled the ‘Big Two’ traits of Social Cognition, although competence and dominance may be less closely related than are warmth and trustworthiness, as previously discussed (Imhoff et al., 2013; Oldmeadow et al., 2013; Oliveira et al., 2019, 2020; Sutherland, Oldmeadow, et al., 2016; Walker & Vetter, 2016).

**FIGURE 3:**
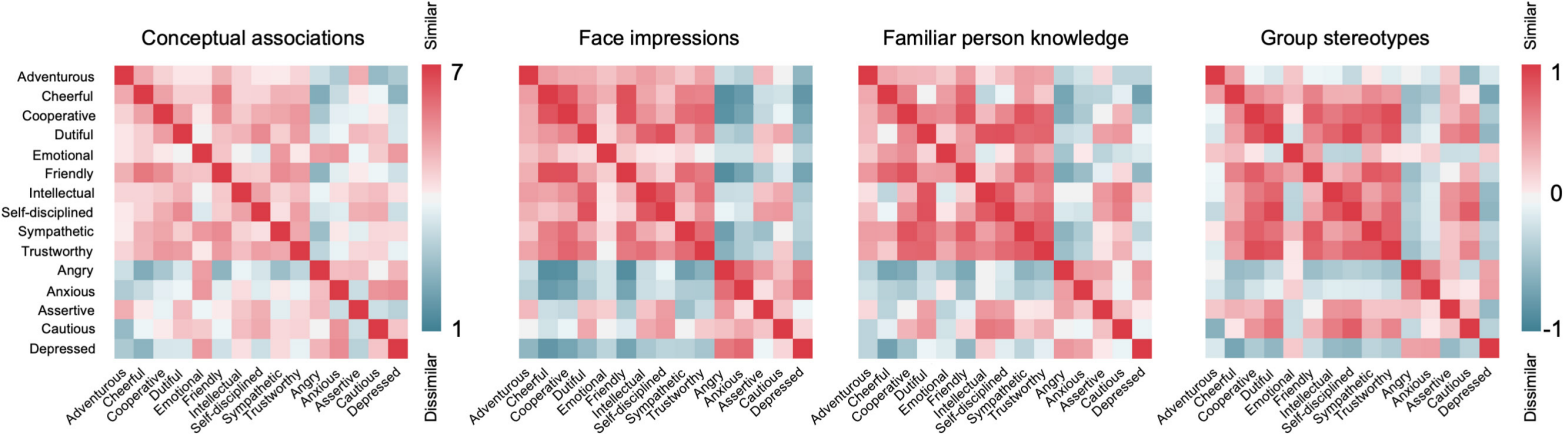
Trait inferences across social cognition mirror conceptual knowledge. Matrices depict pairwise similarity values between each trait pair (plotted from dissimilar (blue) to similar (red) for conceptual impressions, impressions made from faces, impressions of known people and stereotypes of groups. Matrices are sorted by a k‐means cluster solution underlying the conceptual trait space matrix, to intuitively depict their similar structure. Each matrix was collected from a distinct task, set of stimuli and set of participants, yet their similarity shows that conceptual trait space (*n* = 116) is largely reflected in social perceptual trait spaces across domains. The matrices are highly correlated (Spearman *r* = .74 and above; excluding data on and above the diagonal, as the matrices are symmetrical). Data from Stolier et al. (2020), Nature Human Behaviour

Also compelling is the finding that individual differences in conceptual impressions (i.e. with purely verbal stimuli) reflect individual differences in impressions of faces, as measured by representational similarity analysis (Stolier et al., 2018). That is, people who have formed strong conceptual associations between friendliness and intelligence (for example) also show higher overlap in these impressions as judged from faces. These findings demonstrate the potential for top‐down social information to affect facial impressions and highlight the tight link between conceptual social inferences and perceptions of visual cues from faces themselves (Figure 3). This link is perhaps to be expected because the face is simply one source of evidence used to achieve the goals of interpersonal perception and interaction (McAleer et al., 2014; Morrison et al., 2017). It is therefore important to recognize that the small number of dimensions that seem to underlie impression formation from faces may reflect general organizing principles in human cognition more widely. Indeed, Osgood's Semantic Differential model of general attitudes also has three dimensions, evaluation (good versus bad), potency (strength versus weakness) and activity (high versus low) (Osgood, 1969). These concepts bear resemblance to key dimensions of trait impressions from faces (Sutherland et al., 2013). Importantly, Osgood's model goes far beyond face perception, or even interpersonal attitudes, to encompass cognition more generally. For example, in Osgood's model, a tiger is an attitude object which is represented by most people as bad, strong and active (Osgood et al., 1957).

Further evidence for the idea that impressions are organized by general cognitive principles comes from studies on non‐facial person perception. For example, impressions of voices can be summarized by two dimensions of valence (trustworthiness) and dominance, paralleling two of the main face dimensions (McAleer et al., 2014). Vocal cues to trustworthiness and dominance echo facial ones; for example, masculinity cues dominance for male speakers (McAleer et al., 2014). Moreover, female and male vocal attractiveness diverges, with female vocal attractiveness being more strongly valenced and male vocal attractiveness more influenced by perceived dominance (McAleer et al., 2014), similarly to facial attractiveness (South Palomares et al., 2018). Impressions of bodies also align with valence and agency (dominance) dimensions (Hu et al., 2018), although another study found that only a single valenced factor underpins impressions from bodies (Morrison et al., 2017). It is currently an interesting open question as to how these different sources of information are integrated to serve interpersonal perception, and where this integration occurs in the brain (Young et al., 2020). It will also be important for future theorizing to determine whether impressions are based on domain‐general aspects of cognition (as suggested by the semantic differential model) or specific to person perception (as is perhaps otherwise implicitly assumed).

### Why do we form face impressions?

Given that facial impressions have reasonably low validity (in the sense that a given individual who looks trustworthy may not be trustworthy, Foo et al., 2021), why do we persist in forming and acting on them so readily? Theories of first impressions suggest the reason is that impression formation is nonetheless functional; we form impressions because we seek to predict our social interactions (Fiske et al., 2007; Oosterhof & Todorov, 2008; Sutherland et al., 2018; Zebrowitz, 2005). Moreover, impressions may have enough of a kernel of truth, or at least overlap with other, more accurate judgements, to allow them to persist at a population level even with low overall accuracy (Foo et al., 2021).

Initially, theorizing concentrated on impressions as functional for judging different aspects of threat, important to human survival and likely shared with other primate species (Fiske et al., 2007; Oosterhof & Todorov, 2008). For example, impressions of valence (captured by trustworthiness) may be linked to evaluating whether someone is seen as likely to have good or bad intentions towards you, whereas dominance is an evaluation of whether they can carry out those intentions. In most current models of impressions, impressions of intentions (and often, associated threat) therefore play a key role. Indeed, as noted already, impressions related to good or bad intentionality nearly always emerge as the main source of variance in a wide variety of studies, with different traits, varied face samples and with multiple analytic methods (Fiske et al., 2007; Lin et al., 2021; Oosterhof & Todorov, 2008; Sutherland, Oldmeadow, et al., 2016; Walker & Vetter, 2016; Wolffhechel et al., 2014). In some models, impressions of age and attractiveness are also important and linked to physical health (Lin et al., 2021; Sutherland et al., 2013). Judging others on their health may have utility for sexual selection (Rhodes, 2006), is important to avoid current illness (Jones et al., 2022), and may also be linked to age‐related stereotyping (Sutherland et al., 2013).

More recently, attention has also been paid to the role of first impressions in wider social behaviour, including for affiliation (Sutherland et al., 2018; Zebrowitz & Franklin, 2014), romance (South Palomares et al., 2018) and the nurturing of children (Collova et al., 2019). Collova et al. (2019) argued that the main dimensions underlying variance in first impressions should vary across different social groups to the extent that social goals differ with these groups. Indeed, key impressions of children by adults appear to be niceness and shyness, which correlated highly with valence (and trustworthiness) but not at all with dominance (Collova et al., 2019). Importantly, this finding supports the idea that impressions are functional (Oosterhof & Todorov, 2008) and dynamically adapted to the social context (Stolier et al., 2017). These newer accounts are important because they widen the focus of impression formation beyond simply judging threat.

#### Overgeneralization

Face impressions are powerful because they represent our desire to reduce uncertainty when interacting with strangers. This need to predict what strangers might do may have arisen relatively recently in our evolutionary past, when humans stopped living in small communities and began increased interactions with strangers (Mileva et al., 2019; Todorov, 2017). It is likely that impressions of strangers bear a strong relation to cues to social signals from familiar others, which are then mis‐applied to unfamiliar faces. In other words, impressions have bootstrapped (or overgeneralized) from other, older mechanisms. In line with this theory, impressions modelled from observers' responses to one face identity can be generalized to new images of both the same *and* new identities (Mileva et al., 2019). Moreover, modelling work has also shown how the encoding of cues that are directly related to facial impressions can be an emergent property of training networks in familiar face recognition (Kramer et al., 2017; Parde et al., 2019). Thus, there is evidence that impression formation (of unfamiliar faces) can be an emergent property of other aspects of (familiar) face recognition. It would be interesting to test the reduction of uncertainty theory in small‐scale, non‐industrialized non‐WEIRD cultures, where interactions with strangers are less common, or in situations where uncertainty is experimentally controlled. If impressions are formed due to a need to reduce uncertainty, then we predict that people in smaller scale societies, or after experimental uncertainty reduction, would show reduced spontaneous impression formation.

In one key overgeneralization process, ‘temporal extension’, human observers misattribute stable characteristics from a specific encounter with an unfamiliar person (Secord, 1958). For example, someone who expressing anger, rather than being viewed as experiencing a temporary emotional state, is viewed as having an untrustworthy personality; an idea that has been further developed by Zebrowitz and her colleagues to cover emotional overgeneralisation from neutral faces that merely happen to resemble emotional expressions (Montepare & Dobish, 2003; Zebrowitz et al., 2003, 2010). Interestingly, the same tendency can be seen when Chinese and British people are asked to describe strangers' faces from naturalistic photographs; they tend to use trait level descriptions (‘friendly’) rather than emotional labels (‘happy’) (Sutherland et al., 2018). This tendency to generalize to traits may be related to observations described in theories from other branches of psychology, including the fundamental attribution error (Todorov, 2017) and mind‐mindedness (Zeegers et al., 2017). Mind‐mindedness, as described in developmental psychology, for example, refers to the tendency for caregivers to differ in the extent to which they read intentions into their child's behaviour (Meins, 1997). We predict that individuals who are higher on mind‐mindedness will also be more likely to read enduring traits into images of faces.

Inferring enduring traits from emotional states or resemblance to emotional expression is not the only process by which people overgeneralize. Arguably, the well‐known attractiveness halo effect, which is the tendency for people to attribute other socially desirable traits to attractive individuals (Dion et al., 1972), is also an over‐generalization effect (Zebrowitz & Collins, 1997). Overgeneralization from social groups, or stereotyping, also appears to influence impression formation from faces (Oldmeadow et al., 2013; Zebrowitz et al., 2010). Cues to physical maturity which differ between adults and children are also overgeneralized to judge ‘baby‐faced’ and ‘mature‐faced’ adults (Zebrowitz & McDonald, 1991).

Strikingly, perceivers also infer traits from facial cues based on past social experiences (‘*parataxis’*; FeldmanHall et al., 2018; Secord, 1958). For example, Lewicki (1985) found that, after initial exposure to a friendly and warm target, participants were more likely to choose a face more closely resembling this target person as being ‘kinder and friendlier’ than a control face (see also Verosky & Todorov, 2010). A recent twin study suggested that individual differences in impression formation may be especially shaped by unique individual experiences, likely reflecting this process (Sutherland, Burton, et al., 2020; see also section ‘Origins in individual learning’). It will be important to test this learning account further using personally familiar faces.

Finally, facial features with functional or metaphorical significance can lead to corresponding trait inferences (Secord, 1958), as was common practice among the physiognomists (Bruce & Young, 1986). For instance, since the lips function for talking, people with large mouths may be seen as talkative, by metaphorical association. Indeed, many traits are themselves metaphors, such as ‘warm’, ‘cold’, ‘strong‐minded’, ‘bright’, ‘leonine’ or ‘foxy’. These possibilities have yet to be thoroughly systematically tested, although they were the basis for some of the very first empirical studies on face‐based first impressions (Thornton, 1944; Zebrowitz et al., 1996), and one study has found preliminary evidence for metaphorical associations from animal to human faces (Zebrowitz et al., 2011).

In sum, impressions may represent the outcome of more general, functional, and powerful mechanisms that are (mis)applied to infer traits from strangers. Like any stereotypes (Macrae & Bodenhausen, 2000), they function to help people predict their social environment by modelling the world ‘as is’. Thus, for example, it is helpful to a social observer to know that contingencies between behaviour and gender exist, regardless of the wider (and often deeply unfair) underlying societal reasons why men appear powerful, and women are likely to appease. Face‐based impressions, like any other stereotype (Macrae & Bodenhausen, 2000), help us to navigate complex social worlds with efficiency and ease. Taken together, this limited functionality is enough to promote the use of impressions despite our best attempts not to ‘judge a book by its cover’.

### The origins of facial impressions

Theories of facial impressions have highlighted the importance of cultural and individual learning (Oh et al., 2020; Over & Cook, 2018; Stolier et al., 2017; Sutherland, Young, et al., 2015; Zebrowitz et al., 2007) as well as drawing attention to the role of evolution in shaping face preferences at a population level (Oosterhof & Todorov, 2008; Rhodes, 2006; Sutherland et al., 2013; Zebrowitz et al., 2003). These accounts are not mutually exclusive, and all three aspects are useful in understanding how and why impressions are formed (Sutherland, Collova, et al., 2020).

#### Origins in cultural learning

Cultural learning clearly affects facial impressions. For example, glasses emerge spontaneously as a cue to impressions of intelligence in a data‐driven model (Sutherland et al., 2013); experimental studies also confirm that glasses cue intelligence (Leder et al., 2011). People also readily form impressions of modern occupational stereotypes, such as a nurse or banker, from facial cues (Imhoff et al., 2013; Oldmeadow et al., 2013). These findings rely on cues or impressions that are highly novel in evolutionary terms, so the trait‐cue mappings are presumably learnt through cultural experience.

**FIGURE 4:**
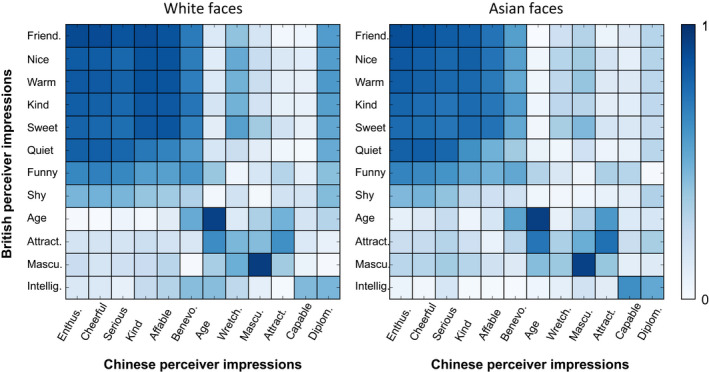
Heatmaps depicting degree of agreement in impressions across British (horizontal) and Chinese (vertical) perceivers judging White and Asian faces. Blue, *r* = 1, white, *r* = 0. Impressions depict the most frequently mentioned traits in each culture and are sorted by their contribution to British and Chinese models. The clustering pattern directly reflects dimensions emerging from more complex analyses. A strong cluster of intentionality traits can be seen, a moderate cluster for youthful‐attractiveness traits, and a small cluster for capability traits. British: Friend. = Friendly, Nice, Warm, Kind, Sweet, Quiet, Funny, Shy, Age, Attract. = Attractiveness, Mascu. = Masculinity, Intellig. = Intelligence. Chinese: Enthus. = Enthusiastic, 热情, Cheerful = 开朗, Serious = 严肃, Kind = 和善, Affable = 和蔼, Bene. = Benevolent, 慈祥, Age = 年轻人/老年人/, Wretch. = Wretched, 猥琐, Mascu. = Masculinity, 女性化的/男性化的, Attract. = Attractiveness, 吸引力, Capable = 干练, Diplom. = Diplomatic, 圆滑. Data re‐analysed from Sutherland et al. (2018), *Personality and Social Psychology Bulletin*

A widely‐used approach to issues of cultural learning is to compare responses across participants with different cultural backgrounds. There is a surprisingly high overall correspondence in impressions across different cultures (Lin et al., 2021; Sutherland et al., 2018; Walker et al., 2011; Wang et al., 2019), even when very different groups are compared (Zebrowitz et al., 2012). For example, Figure 4 reanalyses data from a cross‐cultural experiment with Chinese and British perceivers: there are more similarities than differences across these two cultures for both Asian and Caucasian faces. A landmark study showed that impressions show high agreement across culture for both own‐and other‐race faces between the Tsimane’ people of Bolivia and Americans tested in the US (Zebrowitz et al., 2012). This finding is especially remarkable because the Tsimane’ people are a culturally isolated group, who have no access to global media and minimal contact with Westerners. Recently, the inaugural Psychology Accelerator multi‐lab project study investigated the replicability of the Oosterhof and Todorov (2008) valence by dominance dimensional model across 11 world regions (Jones et al., 2021). This study found remarkable similarity across cultures when replicating the original principal components model. However, some differences were noted across the cultural samples when an exploratory factor analysis was used with an oblique rotation. Why differences emerged in these data is not yet clear, as other studies have not found comparable analytic choices to change overall conclusions across culture (Lin et al. 2021, Sutherland et al., 2013, 2018), nor are differences found when the original Oosterhof and Todorov (2008) dataset is re‐analysed with an oblique model (Jones et al. 2021). Importantly, the patterns of correlations between traits (i.e. the raw data entered into a dimensional analysis), appear very similar across culture (see also Figure 4), thus it remains to be seen to what extent these analytical differences represent meaningful cultural dialects (Todorov & Oh, 2021). Intriguingly, another reanalysis of the Jones et al. (2021) dataset finds that impressions may differ more across individual perceivers than through cultural variation (Hester et al., 2021).

However, a limitation with many of these cross‐cultural studies is that traits were pre‐specified and were chosen as important from Western culture (i.e. they are etic studies, or ‘from the outside’). Emic studies (‘from the inside’) of cross‐cultural impressions from faces are less common. As an exception, Sutherland et al. (2018) found that free descriptions of faces were similar when Chinese and British participants were asked for their unconstrained impressions. Studies of the Trobriand people in Papa New Guinea although not testing for impressions per se, are also suggestive in that people in this culture depict facial representations of threat, albeit with different facial cues (Crivelli & Fridlund, 2018) and show similar impressions of the valence of faces, although not individual emotion labels, to Western judges (Crivelli et al., 2017).

Despite these broad cross‐cultural similarities, at a more detailed level, trait impressions do also appear to show some cultural differences. Impressions of capability‐related traits (dominance, competence or intelligence) seem particularly subject to cultural influences, whereas intentionality‐related traits appear to be more similar across culture (Sutherland et al., 2018; Wang et al., 2019). Moreover, perceivers judging other‐culture faces have been found to take longer (Walker et al., 2011) and show less agreement than own‐culture judges (Zebrowitz et al., 2012), suggesting the presence of cultural ‘dialects’. Indeed, smiling is seen as intelligent in some countries (e.g. Germany), but as a sign of stupidity in others (e.g. Japan and Russia: Krys et al., 2013). Although most cultures view smiling as a sign of honesty, interestingly, this association is also weaker in countries with higher corruption indices, suggesting that people either fail to learn this cue link as strongly, or alternatively, have learnt to ignore or override this cue (Krys et al., 2013).

We have suggested that face‐based impressions show variform universality, so that the underlying function of impressions will be more similar across cultural contexts than specific facial cues to these impressions, which will vary as a function of their utility in that context (Sutherland et al., 2018). For example, glasses can presumably only cue intelligence in cultures where glasses are regularly (but not universally) worn. This theory also predicts that capability inferences will be especially contextual, given that capability is specific to a task or goal. Available cross‐cultural data support this idea (Krys et al., 2013). However, this prediction remains to be tested by experimentally manipulating the context rather than relying on cultural differences created outside the lab. Moreover, there are other important cultural contexts which have yet to be systematically investigated, such as social identity groups (e.g. ‘hipsters’, ‘goths’, ‘anime kids’, etc.) and rural versus urban divides. Future research should explore more culture‐specific models, especially with small‐scale, non‐industrialized, non‐WEIRD populations.

#### Origins in individual learning

While cultural learning is relevant to understanding consensual judgements across broad social groups, there are also individual differences in impressions of traits from faces within a culture (Hehman et al., 2017). Although most research to date has concentrated on the consensus agreement across observers, their ‘shared taste’, it turns out that around half of the variation in facial impressions is driven by individual differences between observers, their ‘private taste’ (Hehman et al., 2017). This result builds on similar findings for facial attractiveness (Germine et al., 2015; Hönekopp, 2006). Interestingly, just as intentionality‐related traits appear to be more stable across culture than capability‐related traits, the same is true for individual differences in impressions made by observers within a culture (Hehman et al., 2017; South Palomares et al., 2018; Sutherland, Rhodes, et al., 2020). However, the same dimensions of impressions exist at the individual perceiver level as well as at the group level, suggesting that these dimensional models can also apply to individual perception (Lin et al., 2021; Sutherland, Rhodes, et al., 2020).

What are the origins of these individual differences in impressions? Interestingly, two recent large‐scale twin studies suggest that idiosyncrasy in facial judgements of trustworthiness, dominance and attractiveness is largely due to unshared environmental influences, not inherited genetic variation (Germine et al., 2015; Sutherland, Burton, et al., 2020). That is, observers differ uniquely in their impressions based on their specific environment. Multivariate twin modelling further showed that the strongest effects are for specific, personal environmental factors, such that the variance across different impressions is largely dissociable (Sutherland, Burton, et al., 2020). In other words, the environmental context matters when learning idiosyncratic impressions.

What mechanisms can explain this learning? Secord and Jourard (1956) suggested that impressions of strangers could be influenced by overgeneralization of traits from familiar others who look physically similar, a process they named parataxis. We propose three broad aspects to parataxis: statistical learning, familiarity, and learning from specific social encounters.

Statistical learning of trustworthy impressions, for example, refers to the phenomenon whereby facial cues that are encountered more often lead to higher trust (Dotsch et al., 2016). This statistical learning may rely on perceptual adaptation, a process of perceptual recalibration that has classically been shown to affect attractiveness (Langlois & Roggman, 1990; Rhodes & Tremewan, 1996). For example, more average faces are seen as attractive (Langlois & Roggman, 1990). The exact links between adaptation and statistical learning are not yet understood, but what is clear is that everyday perceptual experience can reshape the perceptual system, determining who looks trustworthy, attractive or dominant.

DeBruine (2005) provided evidence for familiarity as a cue to impressions, showing that individual differences in trustworthiness perception can be driven by kinship cues, so that people trust others who look like their family members. Verosky and Todorov (2010) subsequently showed that this familiarity benefit extends to impressions of strangers who also look like the self. Familiarity may also partially mediate trait impressions based on racial or other stereotypes (Secord & Jourard, 1956; Zebrowitz et al., 2007).

Learning from specific social encounters also guides facial impression formation. Hassin and Trope (2000) theorized that people can ‘read into faces’ the traits of similar‐looking individuals they have come into contact with previously (see also Secord, 1958). Indeed, when people play financial trust games, they base their partner choices on those who resemble trustworthy people encountered in previous interactions, and avoid partners who resemble untrustworthy others (FeldmanHall et al., 2018; see also Lewicki, 1985; Verosky & Todorov, 2010). This account is most clearly supported by multivariate twin modelling work, which shows that unique rather than shared environments are most important in shaping individual variability in impressions, as are specific contexts (Sutherland, Burton, et al., 2020).

Aside from individual social learning, other candidate mechanisms that might underlie individual differences in impressions include personality traits (for example: individuals who are higher on dominance themselves, are less able to discriminate dominant facial features: Mattarozzi et al., 2015; Watkins et al., 2010), prejudicial attitudes (Dotsch et al., 2008) and/or motivation (Jones et al., 2011). We might further predict that individual differences in impressions would also be driven by an individual's frequency of interaction with strangers and/or their tolerance for social uncertainty if impressions of strangers have developed from perceptual mechanisms adapted for familiar (personally known) faces, in order to reduce uncertainty (Todorov, 2017).

Finally, there are also stable individual differences in the extent to which people believe their facial impressions are accurate (Jaeger et al., 2019; Livingston, 2001; Suzuki et al., 2017). Such physiognomic beliefs are related to the belief that biology is relatively fixed and immutable, and belief in a just world (perhaps reflecting the idea that ‘people get the face that they deserve’) (Suzuki et al., 2017). Importantly, people with stronger physiognomic belief show an increased tendency to base their decisions on superficial face judgements (Jaeger et al., 2019; Livingston, 2001; Suzuki et al., 2017). An interesting question for future research is therefore to explore the links between individual differences in physiognomic belief, behaviour and impressions themselves.

#### Evolutionary origins

Although we clearly learn impressions from our surroundings, noting that our surroundings are biased in turn begs the question of where these biases come from. To answer this question, theorists have also pointed to the evolutionary origins of impressions (Oosterhof & Todorov, 2008; Zebrowitz & Collins, 1997). Evolutionary models have attempted to explain the broad patterns of similarity in impressions across culture, species and in development. For example, as noted previously, dimensions of impressions appear across culture, and impressions are also (partly) consistent across observers within a culture. In a similar way, hypotheses concerning the evolutionary basis of stereotype representation have been used to explain why stereotypes appear to show some universal patterns across culture, albeit with contextual differences in specific stereotype content (Fiske et al., 2007).

There are also links between social perception in humans and other primates which are suggestive of an evolutionary basis to impression formation (Costa et al., 2018). Macaque monkeys show implicit preferences for trustworthy human faces, as demonstrated by longer time spent looking at trustworthy than untrustworthy faces in a preferential looking paradigm (Costa et al., 2018). Vice versa, humans are able to distinguish personality traits and health cues from chimpanzee faces, with judgements by strangers corresponding with zookeeper reports of the chimpanzees' behaviour (Kramer et al., 2011; Kramer & Ward, 2012). Human impressions of Barbary macaque faces also predict human intentions to approach or avoid particular individual monkeys as well as actual macaque behaviour (Clark et al., 2020). Taken together, these results are supportive of a shared primate facial signalling system.

Developmental studies have also shown a surprisingly high correspondence between impressions of children and adults (Caulfield et al., 2014; Cogsdill et al., 2014; Collova et al., 2020; Ewing et al., 2019). Children's impressions can even predict the results of real political elections (Antonakis & Dalgas, 2009). Similarly, children's impressions also drive their own social behaviour, including financial lending (Ewing et al., 2015, 2019), although not always with fully adult‐like patterns (Mondloch et al., 2019). Most strikingly, even newborn infants show orienting responses towards more attractive faces (Slater et al., 1998, 2000), and very young infants (only a few months old) prefer to look at attractive and trustworthy faces (Jessen & Grossmann, 2016; Langlois et al., 1987). Event‐related potentials recorded in seven‐month old infants also distinguish between trustworthy and untrustworthy faces (Jessen & Grossman, 2019). Overall, although there do appear to be developmental trends, such that impressions do not fully mature until around age 10 onwards, children's impressions are in many ways similar to those of adults (Siddique et al., 2022). One important aim of future research will be to map the developmental trajectory of face impressions within a well‐powered longitudinal study. It will also be important to understand which specific cognitive (e.g. attention, motivation), perceptual (e.g. face encoding, identity, emotion recognition) and social (e.g. media, stereotyping, learning) mechanisms underly developmental trends.

Evolutionary‐based accounts explain the early emergence of impressions in development, as well as the close links between different human and other primate facial impression signalling, because they link facial impressions to adaptive functioning. For example, impressions of threat can be based on cues to anger that denote a threatening intention, or physical strength and maturity, which denote capability in carrying out a potentially threatening intention (Oosterhof & Todorov, 2008; Zebrowitz et al., 2003). Attractiveness perceptions may also be adaptive, because attractiveness can signal the current or reproductive fitness of the people around us (Foo et al., 2020; Rhodes, 2006). Although links between measures of immunocompetence and appearance are debatable, there are certainly reasons to think that attractiveness is related to current physical health (see Jones et al., 2022 for a discussion). Indeed, suggesting that someone looks ill is not usually taken as a compliment. Note that the cues themselves need not be especially accurate for inferring traits, rather, as previously discussed, they may reflect processes of over‐generalization from more fundamental adaptive processes (section ‘Origins in cultural learning’). By analogy, first impressions are akin to allergies, which also represent highly functional systems (for parasite avoidance) that were protective in our past or for some specific contexts, but become unhelpful when overgeneralized to modern environments or misapplied to the wrong context (Foo et al., 2020). For example, although it is adaptive (and accurate) to be able to discriminate between children and adults, people also overgeneralize to adults who happen to look more or less mature, in often problematic ways (Zebrowitz & McDonald, 1991).

Importantly, an account which includes evolutionary influences need not imply that facial cues must be innately ‘hard‐wired’, that learning has no effect, or that impressions aren't biased. Moreover, any selection pressure on preferences might be specific to faces, or might drive general information‐processing mechanisms, or both (Quinn et al., 2008; Rhodes, 2006). While it is probable that both specific and general processes affect newborn orienting towards faces, it is notoriously difficult to conclusively show that cues are truly ‘innate’, given that even newborns can show rapid perceptual learning from facial cues in a single test session (Slater et al., 2010). We therefore suggest that, even if a tendency to orient towards faces and/or a propensity to form impressions is inherited, specific associations between faces and traits are likely learnt early in development. Indeed, only a few different images of familiar faces need to be shown before perceptions generalize to impressions of strangers (Mileva et al., 2019); thus the newborn perceptual system could learn face‐trait associations relatively easily. Moreover, many of the underpinning cues are also formed in development; for example, social group preferences for own‐race faces, which influence impressions including trustworthiness, appear in the first few months of life (Slater et al., 2010). Preferences for faces of the same gender as the primary caregiver also appear early in development (Bushnell et al., 2011; Quinn et al., 2002). A period of perceptual narrowing is similarly found for facial recognition (Slater et al., 2010), an ability that shows significant heritability (Shakeshaft & Plomin, 2015; Wilmer et al., 2010). Overall, these face‐tuning processes also show parallels with the exquisite attuning to language that occurs in the first year of life (Slater et al., 2010). It will be well worth investigating whether face‐based impressions show similar early development and/or establishing if there are other sensitive periods in development (e.g. in puberty).

Note that evolutionary models do not offer the only explanations as to why cultures can be similar. Although less studied, cumulative cultural evolution (the sharing of knowledge over generations: Hutchison & Martin, 2015) and ecological factors can also explain cross‐cultural similarities as well as differences. For example, Scott et al. (2014) found that masculine‐looking men looked more aggressive than feminine‐looking men across many different cultures. Interestingly, these impressions became stronger as cultures became more industrialized, but cultural linguistic similarity and health did not predict cultural differences, suggesting that the ‘visual diet’ of faces encountered is important in consolidating these impressions (i.e. witnessing masculine men being more aggressive). Of course, ecology likely cannot explain how all impressions are learnt from the surrounding environment, given that, as already noted, the accuracy of impressions tends to be low (Foo et al., 2021; Todorov et al., 2015). Nevertheless, a careful unpacking of which cross‐cultural variables predict different impression mechanisms will greatly help the field move beyond simplistic dichotomies.

Understanding to what degree different facial impressions are driven by predispositions to certain cues, perceptual narrowing or longer‐term socio‐cultural learning generates exciting new empirical questions for the field to answer (Box 2). It will also be important to specify the developmental stages at which different facial (or other) cues contribute towards impressions. We urge clarity of theorizing alongside this research: rather than focusing on an artificial separation between ‘nature’ and ‘nurture’, we note that developmental change in individual organisms is also crucial to evolutionary change across generations and modern evolutionary accounts suggest that ‘nature’ and ‘nurture’ are best conceptualized as iterative aspects of the developmental process (Honeycutt, 2022). Development of human behaviour is itself best characterized as a self‐organizing, probabilistic process, in which patterns emerge and change due to dynamic interactions between both internal and external factors (Lickliter & Honeycutt, 2003): adaptive processes prepare organisms to face environmental challenges, and genes are always expressed in an environmental context, meaning that nature and nurture are inevitably intertwined (Sutherland, Burton, et al., 2020; Sutherland, Collova, et al., 2020). A multitude of different mechanisms likely prepare us to form the social impressions that allow us to be able to trade resources, find romance and put our trust in perfect strangers.

BOX 2 **Critical future research questions**

- To what extent are impressions perceptual and/or cognitive? Can the dimensional structure of facial impressions be experimentally modified based on top‐down stereotyping and/or bottom‐up facial variation?
- How and where in the brain are impressions integrated across modality (from faces, voices, bodies and so on)?
- Are impressions based on mechanisms specific to person perception, or do they reflect domain‐general processing, or both?
- What is the developmental trajectory for facial impressions, especially from infancy through to early childhood? Can newborns distinguish different trait impressions or do they simply track valence?
- What does the structure of facial impressions look like in less industrialized cultural contexts, including non‐WEIRD cultures, and which cues are used?
- Most studies have used an emic approach, investigating traits derived from Western culture when testing across cultures (see Sutherland et al., 2018 for an exception). Do all cultures spontaneously form the same trait impressions from faces?
- Which cross‐cultural variables specifically explain differences across culture? Which specific patterns of linguistic, cultural, ecological, geographical, or genetic similarity predict impression formation similarity?
- What specific mechanisms drive individual observer variation in facial impressions?
- Can social learning explain differences in the extent to which people use their impressions to drive decision making?
- Given that impressions can be shaped by unique social experiences, how easily can they be trained or reshaped? Addressing this question is particularly crucial given the important social consequences of face impressions.

## CONCLUSIONS

Impressions from faces may seem effortless, but building a satisfying account of face‐based impression formation has proved to be anything but easy. In this review, we confronted multiple apparent contradictions. Impression formation from faces can be driven by both perceptual cues and conceptual stereotyping. Moreover, impression formation appears to be surprisingly similar across culture, gender, individuals and type of input, and yet, there is also clear evidence for variability in impressions at each of these levels. Social learning accounts appear highly plausible, but fail to consider deeper patterns in similarity in impression formation. Meanwhile, purely evolutionary accounts fail to acknowledge the effect of the environment on impression formation. Finally, it remains an open question to what extent impressions from faces are best explained by processes specific to person perception rather than domain‐general mechanisms.

We have two main suggestions for the field. In terms of methods, we think it vital that theories of impression formation are derived from experiments using large‐scale databases of naturalistic, unposed, face images. Experimental paradigms must also be able to detect the influences of multiple interacting cues. Psychology has long realized the importance of testing representative groups of participants: we urge that researchers also take the same care in their choice of target stimuli (whether faces, videos or voices). We see exciting possibilities for ‘many‐lab’ creations of face stimuli databases to help overcome difficulties in recruitment. Multi‐lab recruitment could help generate more diverse face samples, including non‐WEIRD, non‐White and non‐neurotypical or otherwise underrepresented groups. Relatedly, we need more data with diverse perceiver groups, including from non‐industrialized regions. Especially, there may be illuminating differences between production and foraging societies, or between societies with large or small social networks (Ember, 2020; Hill et al., 2011) which could help pin down the mechanisms behind impression formation.

In terms of theory, we suggest that theorizing in the field needs to move beyond simple dichotomies to reconcile these apparent contradictions. First, it will be important not to invoke straw men: social learning, stereotyping and evolutionary accounts are not mutually exclusive (Hutchison & Martin, 2015). Here, we suggest that face‐based impressions are themselves a kind of stereotype. After all, outside of the lab, stereotypes are themselves usually driven by visual or other sensory cues, rather than verbal or conceptual information (Quinn & Macrae, 2011). Second, it may help us to think about underlying mechanisms in terms of degrees, rather than kind. Some aspects of impression formation may be more driven by social experience than others or be particularly context‐dependent (e.g. judging someone's intelligence by the glasses they wear rather judging their warmth from their smile). It is also inadequate to only link evolutionary origins to similarity across groups or species and social learning to differences, given that evolutionary change often involves divergence (e.g. via genetic drift), and cultural and ecological factors can also lead to similarity. Finally, theorists need to pay close attention to which aspect of impression formation they are investigating. A first impression based on a single glance of a highly‐controlled face as studied in a fast‐paced EEG paradigm (Swe et al., 2020), for example, does not need to draw on the same processes as a judgement task that allows one to explicitly compare faces across social groups (Sutherland, Oldmeadow, et al., 2016). It will be critical to consider specific underlying mechanisms, whether cognitive or perceptual, when addressing impression formation phenomenon.

To this end, we outline a set of future research questions for the field, to help build this more sophisticated understanding. Our models need to allow for a multiplicity of cues to impressions, both facial and non‐facial, they must account for consistency in impression formation while allowing for cultural and individual variation, and they should situate the process of impression formation in our primate ancestry as well as our current and past societal environments. A satisfying account of impression formation will only emerge by considering these different theoretical strands together to explain how we form these impressions from faces, so powerful in guiding our daily lives.

## AUTHOR CONTRIBUTIONS

**Clare AM Sutherland:** Conceptualization; writing – original draft; writing – review and editing. **Andrew W Young:** Conceptualization; writing – review and editing.

## CONFLICTS OF INTEREST

There are no competing interests to declare.

## Data Availability

None.
